# Effectiveness of a tailor-made intervention for pregnancy-related pelvic girdle and/or low back pain after delivery: Short-term results of a randomized clinical trial [ISRCTN08477490]

**DOI:** 10.1186/1471-2474-7-19

**Published:** 2006-02-27

**Authors:** Caroline HG Bastiaenen, Rob A de Bie, Pieter MJC Wolters, Johan WS Vlaeyen, Pieter Leffers, Foekje Stelma, Janneke M Bastiaanssen, Gerard GM Essed, Piet A van den Brandt

**Affiliations:** 1 Department of Epidemiology, Maastricht University, P.O.Box 616, 6200 MD Maastricht, The Netherlands; 2 Department of Obstetrics and Gynaecology, University Hospital Maastricht, Maastricht, The Netherlands; 3 Department of Medical, Clinical and Experimental Psychology, Maastricht University, The Netherlands; 4 Department of Physiotherapy, Hogeschool Zuyd, Heerlen, The Netherlands

## Abstract

**Background:**

For the moment, scientific evaluation of programs on treatment of pregnancy-related pelvic girdle and/or low back pain after delivery is hardly available with only one study with a positive result, suggesting uncertainty about the optimal approach. Investigators draw particular attention to biomedical factors but there is growing evidence that biopsychosocial factors appear to be even more important as a basis of an intervention program.

**Methods:**

We studied the effectiveness of a tailor-made program with respect to biopsychosocial factors (intervention group) in women with pregnancy-related pelvic girdle and/ or low back pain versus usual care based on a pain contingent basis (control group) shortly after delivery in a randomized controlled trial. Women with severe complaints shortly after delivery were selected from a longitudinal prospective cohort study (n = 7526), aimed at pregnancy-related pelvic girdle and/or low back pain in the Netherlands. A concealed block randomization was performed after collecting baseline data. Researchers were blinded to treatment assignment. Outcomes were evaluated within the domains of the biopsychosocial approach. Primary outcome concerned limitations in activities (RDQ). Follow-up measurements were performed 12 weeks after delivery.

**Results:**

Since May 2001 until July 2003, 869 women out of the cohort made a request for treatment by a physiotherapist, 10 days after delivery. Because of a quick recovery in two weeks time, we included only 126 women three weeks after delivery. There was a statistically significant and clinically relevant difference in improvement on the primary outcome (RDQ) between the two groups in favor of the experimental intervention.

**Conclusion:**

The results favored the hypotheses. Women's worries about their condition were major targets in the experimental intervention. The prognosis after delivery, especially in de first weeks, turned out to be favorable.

## Background

Effective therapy depends to a large extent on the adequate interpretation of early symptoms and the outcome of the diagnostic process [1]. These outcomes make a positive contribution to the communication between caregiver and patient and have a meaningful influence in goal setting for intervention. The research field of pregnancy-related pelvic girdle and/or low back pain is characterized by considerable differences of opinions about etiology, diagnosis and prognosis. The controversies relate to factors eliciting pain [2] and prognostic factors such as the interpretation of pain at the symphysis [3,4], the question whether pelvic girdle pain is a syndrome separate from low back pain [4,5] and the importance of questions about limitations in activities [6].

Also on behalf of historical factors, pregnancy-related pelvic girdle pain is approached as a syndrome separate from low back pain outside pregnancy in the Netherlands. A pain contingent approach focused on biomedical elucidations is firmly entrenched among physiotherapists and patients. Currently, the intervention program for this group of patients in the Netherlands aims at increasing disease-specific education and encouraging compliance with prescribed exercises and a pain contingent regime of relative (bed) rest and avoiding and limiting several day-to-day activities such as using the stairs, bending, twisting, lifting and cycling. Therapists rely heavily on reported pain duration and intensity during goal setting for intervention. Moreover, the approach of a physiotherapist more often includes an exercise program to address the muscle imbalance and alignment of the pelvic girdle [7,8]. However, scientific evaluation of programs on treatment of pregnancy-related pelvic girdle pain after delivery is hardly available with only one study focusing on specific stabilizing exercises [8] with a positive result. This suggests uncertainty about an optimal approach. Furthermore, the majority of the studies show methodological flaws [9].

At the moment, several investigators in this field draw particular attention to biomedical factors but there is growing evidence that important prognostic problems such as biopsychosocial factors appear to be even more important as a basis in an intervention program [10,11]. In this study, we refer to the International Classification of Functions (ICF)[12] as a basis for describing and better understanding of pregnancy-related pelvic girdle and/or low back pain. The ICF describes functioning and disability as an interactive process. The biomedical model (as defined by the WHO) [12] views disability as a problem of the person, directly caused by the disease, which requires medical care provided in the form of individual treatment by professionals. In the biopsychosocial approach a person is seen as a system integrating biological, psychological and social dimensions. The approach takes into account prognostic factors that may influence recovery and emphasizes the role of psychological and social factors in the development and persistence of symptoms and limitations in activities. The biomedical aspects have to be required within the biopsychosocial approach rather than presented as something separate and apart.

We performed a double-blind randomized controlled trial in primary care to determine the effectiveness of a tailor-made program with respect to biopsychosocial factors (intervention group) for women with pregnancy-related pelvic girdle pain after delivery versus usual care on a pain contingent basis (control group). The trial is embedded in a longitudinal, prospective cohort study [13], which studies the prevalence, etiology, severity and prognosis of pregnancy-related pelvic girdle pain until one year after delivery. The trial is designed and reported according to the Consolidated Standards of Reporting Trials (CONSORT) statement [14] and is registered in the International Standard Randomised Controlled Trial Number Register [ISRCTN08477490]. Because of the relatively unknown etiology and the lack of an all-embracing definition, we used an extensive description of pregnancy-related pelvic girdle pain including all women who experience some form of pregnancy-related pain in the lower back and/or pelvic girdle originated in the musculoskeletal system.

The present article describes the short-term results. The study design has already been described elsewhere [15]. Long-term results until one year after delivery and costs are reported separately. We found it relevant to report the short-term results separate because we want to evaluate the influence of a possible reduction of pain after delivery on the number of women of the whole cohort that want be to referred to a physiotherapist during pregnancy. Some studies [3,16-18] noted a high prevalence during pregnancy and a considerable drop shortly after delivery. Maybe there are fewer women that want to be referred to a physiotherapist after delivery than during pregnancy.

## Methods

### Recruitment and informed consent

The medical ethics committee of the Maastricht University Hospital approved the intervention and cohort studies which were performed in the Southeast of the Netherlands. Midwives and gynecologists recruited the women during early pregnancy (10–14 weeks). Women were included in the cohort if they were at least 18 years old, pregnant and well versed in the Dutch language (n = 7526). Women were given written information explaining the aims and contents of the cohort and intervention studies before they decided to participate. Concerning the intervention study they were told that according to current knowledge, the two investigated treatment options are considered to be equally effective. The moment of inclusion for the intervention study was about three weeks after delivery. An individual woman entered the intervention study after signing informed consent for both the cohort and the intervention studies during early pregnancy and meeting the in-and exclusion criteria of the intervention study shortly after delivery. A basic principle was that inclusion criteria must have a meaningful influence in goal setting for treatment. Women were included when having pain in the pelvic girdle (including the lower back) with an onset during pregnancy or within two weeks after delivery (cohort data), were restricted in their normal daily activities because of pelvic girdle and/or low back pain and if there was a delay in recuperation (unable to participate satisfactorily in housekeeping and care of children because of the complaints under investigation). The severity of symptoms should vary with physical activities and time during the day. In the absence of a clear definition and reference standard to diagnose pregnancy-related pelvic girdle pain, the outcomes of a number of currently used diagnostic classification strategies not only led to different selections of women having complaints, the prognostic importance of these subgroups remained also unclear. Therefore we used an extensive description of pregnancy-related pelvic girdle pain. Women diagnosed with relevant specific pathology (such as nerve root pathology, rheumatoid disorders, carcinoma, obstetric complications) that affects pain and activities of daily life were excluded. Exclusion also occurred in case of severe family-related or psychosocial problems or when a disablement procedure was not yet finished. Final aspects for in-/exclusion were the willingness of a woman to participate in the study or having a clear treatment preference. We only included women who did not indicate such a preference [19].

An experienced research-physiotherapist visited the women at home, about three weeks after delivery. This visit was called for on the basis of a short self-administered questionnaire or initiated by midwives. A positive answer from a participating woman from the cohort or her midwife: "Is an intervention by a physiotherapist necessary?" took a central position in these questionnaires. Preceding a possible home visit, a short history taking by telephone took place about two weeks after delivery. The telephone interview focused on exclusion criteria such as willingness to participate, specific pathology and inclusion criteria such as limitations in daily life caused by pregnancy-related pelvic girdle pain and a delay in recuperation. During the home visit, a standardized history was taken and physical examination to exclude specific pathology was performed. Self-administered questionnaires were used to asses pain, limitations in activities, restrictions in participation, pain-related fear, pain catastrophizing, positive and negative affectivity, depression, expectancy of treatment result and quality of life. The questionnaires contained clear instructions to complete them with no help or support from others. If a woman met the selection criteria, she was informed about the aim and method of the intervention study and if she was willing to participate, the informed consent procedure was completed. The research-physiotherapist collecting the baseline data was trained at performing the measurements in a standardized way and was blinded for treatment assignment.

### Randomization and blinding

Randomization took place after collecting the baseline data and obtaining informed consent. An independent research assistant (unaware of the base line data) carried out the concealed randomization procedure using a random computer-generated list. Block size for the randomization was four. When a woman was allocated to the intervention group, a physiotherapist trained in the experimental intervention practicing in the neighborhood of the woman was contacted and we ensured that treatment could start within one week. Treatment costs were covered for all participants in the intervention group. Women, allocated to the usual care group, were free to choose usual care treatment by a physiotherapist who was not trained in the experimental intervention protocol.

Women were blinded to a certain extent to the allocated intervention option because they were kept naïve of the exact content of both intervention options. Physiotherapists were not involved in the baseline and effect measurements. Researchers dealing with the baseline and outcome data were blinded to the intervention assignments.

### Interventions

#### Experimental intervention

Women, allocated to the intervention group, were referred to a participating physiotherapist in their own neighborhood. These physiotherapists received an educational program about the treatment protocol prior and during the study. All physiotherapists were already experienced and specialized in treating women with pregnancy-related pelvic girdle pain prior to the study. The content of the experimental intervention was based on the latest literature findings. It focused particularly on patient-therapist relationship, education and the promotion of an active life style rather than avoiding activities. Results of interviews with women from the cohort and group discussions with experienced physiotherapists prior to the trial further defined the program[15].

Theoretical concepts of self-management [20,21] and fear-avoidance [22] were integrated in the intervention protocol. We provided an individualized self-management approach of 7–9 sessions for 30 minutes once a week. Standardized information was presented by means of an intervention protocol for the therapists and by booklets [23] for the patients [21,24]. Topics included back and pelvis anatomy, "red flags" indicating a serious medical condition, factors contributing to fluctuations in pain, appropriate pacing of exercise [7] and activity, handling pain flare-ups, cognitive restructuring, some graded exposure techniques [22,25,26], communication and social persuasion. Therapists had to employ simple problem-solving techniques that engaged women in identifying day-to-day problems or limitations related to pelvic girdle an/or low back pain, setting personal goals, brainstorming options for achieving these goals and developing personal action plans. In subsequent sessions, women reviewed their action plans and their progress towards goals and engaged in problem-solving skills concerning difficulties that arose in trying to implement their plans. Information about two opposing behavioral responses of pain-related fear (avoidance and confrontation) was given and a hierarchy of individual fear-eliciting movements and activities was made. Therapists encouraged women in making action plans with respect to graded exposure techniques in specific activities that were avoided. They were explicitly asked not to "label" the complaints in terms of medical diagnostics.

Generally, a time contingent policy was followed in which women set the pace by means of action plans. The expertise of the physiotherapists of the condition in general and of the women about their own specific condition and lives were equally important [27].

#### Usual care

Women, allocated to the usual care group, were free to choose usual care treatment by an experienced physiotherapist who did not followed the educational program about the treatment protocol, guidance by a general practitioner or do nothing. Information about the option chosen by the women was collected by means of questionnaires in the follow-up period. When a woman opted in favor of treatment, the sessions started within one week.

Prior to the trial, detailed information was gathered about the contents of the current treatment options. Part of the information was gathered by means of group discussions with experienced physiotherapists and occupational therapists and interviews on an individual basis with women out of the cohort. The most striking characteristics of the current treatment were the character of the therapist-patient relation and the way of goal setting, focusing on disease management [21]. There was an explicit professional input and an accent on biomedical aspects. A pain contingent regimen of avoiding and limiting several day-to-day activities was considered important. Compliance based on these goals played an important part for the therapists however they often did report problems with it. However, women reported that they were increasingly irritated about the treatment regime after starting the sessions. The regime was too strict and on a number of points not geared to the wishes and concerns of the women. These aspects influenced probably the reported lack of compliance and an unremitting hesitation about a good prognosis. An exercise program focusing on the muscle imbalance and alignment of the pelvic girdle was a standard part of the intervention [7]. Therapists were often highly concerned about their patient's pain themselves. A biomechanical label with highlighting the attention on pain and emphasizing the vulnerability of the spine and pelvis to damage characterized the patient information.

### Contrast between the experimental intervention and the usual care

The contrast between the experimental intervention and usual care was an important issue in this study. Major features that underscore the contrast were the contents of the patient information, character of patient-therapist relationship, pain-contingent versus time-contingent intervention and compliance to a regime of avoiding and limiting activities versus action planning and personal goal setting by the women themselves. Among therapists, the approach of the experimental intervention is not widespread at all in the Netherlands. The participating therapists were explicitly asked not to give any information about the contents of the experimental treatment to therapists who did not participate in the experimental intervention.

### Measurements

#### History taking and physical examination

During the visit at home a standardized history was taken [28] and physical examination was performed. During history taking, questions about on-going pain, its location, intensity and modalities, variation of symptoms with physical activities, radiation into the legs, back pain versus leg pain, neurological signs, deformity, obstetric complications, a case history of low back and pelvic girdle pain prior to this pregnancy and other differential diagnoses. The format of the answers was presented as a dichotomous "yes or no". Demographic characteristics and data about education, work, income, use of tobacco, medication, the onset of pain and functional status during pregnancy were already gathered as part of the cohort study at 14 and 30 weeks gestation period and two weeks after delivery.

After history taking, a short standardized clinical examination program was performed, which included tests of nerve root radiation (exclusion)[28]. The research-physiotherapist filled out the Pain Behavior Scale [29,30].

#### Primary outcomes

Primary domain (Table 1) for improvement of the intervention was limitations in activities. Other important domains were the severity of the main complaint and global feeling of recovery.

Limitations in activities were measured with the Dutch translation of the Roland Disability Questionnaire (RDQ) [31]. We replaced the phrase "because of my back or pelvic girdle pain" to all questions instead of "because of my back".

The Quebec Back Pain Disability Scale (QBPDS) [32,33] was initially also selected [15] but immediately after the start of the data-collection it became clear that the missing value rate on a number of items (4, 11, 12, 19 and 20) was rather high, shortly after delivery. Those items focus on activities as driving a car, throwing a ball, running and lifting or pushing a heavy weight. Performance of those activities was unlikely for most of the women so shortly after delivery and the scoring possibilities left not enough possibilities to answer those items satisfactorily.

Subjective measurements like global feeling of recovery (global perceived effect, GPE) and severity of the main complaints (MC) reflecting a patient-specific approach were also selected. Global Perceived Effect [34] (GPE) was measured by self-assessment on a 7-point scale (1 = completely recovered, 7 = worse than ever). The main complaints (MC) were selected by the women in a standardized approach by selecting three activities, which are an essential and frequently performed part of her everyday life. However, performance of those activities was difficult or impossible because of pelvic girdle complaints at the moment of baseline measurement. The severity of the selected main complaints was rated with Visual Analog rating Scales (VAS). [34,35].

#### Secondary outcomes

Pain was measured with two Visual Analog rating Scales (VAS) of the McGill Pain Questionnaire (MPQ-DLV) [36,37] to record the intensity of pain the last week and day.

The impact on participation and autonomy (IPA) measured person-perceived restriction in participation and autonomy [38,39]. The used subscales were self-care and appearance, mobility, leisure, social relationships and family role. Perceived participation is graded on a 5-point rating scale ranging from 0 (very good) to 4 (very poor).

Fear of movement was measured by the Dutch translation of the Tampa Scale for Kinesiophobia (TSK)[40,41]. We used the TSK and the both subscales "fear avoidance" and "harm". [42,43]

The Short-Form 36 (SF-36) [44] evaluated health status [45]. We used the subscale "general health".

#### Baseline measurements

Pain catastrophizing is measured by the Pain Catastrophizing Scale (PCS)[46,47].

The Beck Depression Inventory (BDI) [48] was used to measure depressive symptoms [49]. It has been argued that the somatic content of the BDI may lead to false-positives among patients with physical problems. Therefore, items associated with weight loss, sleeping disturbance and work inhibition were excluded in this study [50].

To measure the experience of negative affect we used the 14-item Negative Emotionality Scale [51]. To measure positive affect we used the 11-item Positive Emotionality Scale [51]. Both were subscales of the Multidimensional Personality Questionnaire.

Treatment expectancy [52] was measured by means of a 100 mm visual analog rating scale (VAS). The women were asked to what extend they believed that the treatment is beneficial to them.

The Pain Behavior Scale (PBS)[29,30] is an observation scale tapping 8 behaviors that the physiotherapist completes after physical examination. These are verbal complaints, facial grimaces, standing posture, mobility, body language, use of visible supportive equipment and stationary movement.

### Follow-up

Women were asked to complete a follow-up questionnaire (Table 1) at 12 weeks after randomization. Those women who did not return their follow-up questionnaires were contacted by (e) – mail or phone and were asked to continue participation.

### Compliance, other interventions and confounding

The follow-up questionnaire (Table 1) asked all women how many treatment sessions they had followed since baseline measurement. Furthermore, information on contents, satisfaction and which aspects of the (experimental) treatment they found benefit in. Co-intervention, medication, aids, additional medical consumption were also registered.

### Statistical analysis

The baseline status of the study groups was compared with respect to the distribution of all variables known at baseline. For the outcome measures recorded at baseline and follow-up, we computed the mean difference between the baseline and follow-up score for each woman. Differences between group and 95% CI's were calculated for each outcome measure according to the intention-to-treat approach [53]. Primary analysis was done by means of analysis of an independent t-tests (for continuous outcome variables) and chi-square tests (for categorical outcome variables). Longitudinal missing data were substituted with the "baseline carried forward method". In all comparisons between the two treatment options a two-tailed p-value of 0.05 was considered to indicate statistical significance. Analyses were done by using SPSS statistical software, version 12.0 (SPSS, Inc., Chicago)

## Results

### Recruitment

From November 2000 until November 2002, 7526 women were included in the cohort study.

**Figure 1:**
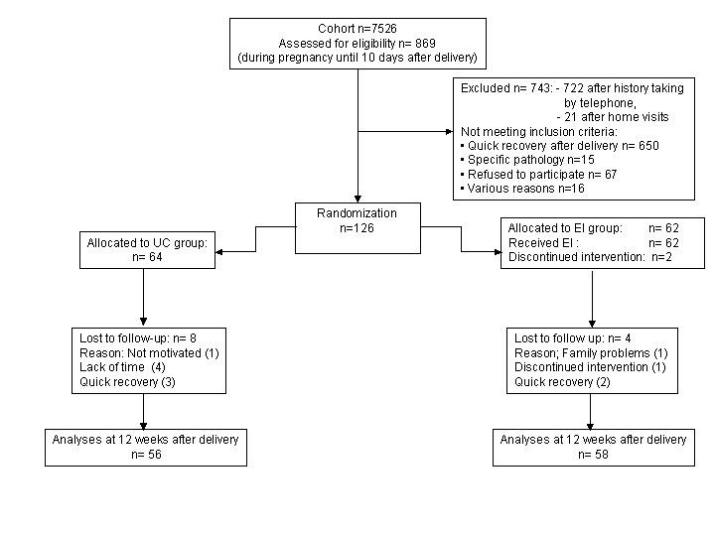
Flowchart of participants through the study.

Results of the self-administrated question: "Is an intervention by a physiotherapist necessary?" resulted in 869 eligible participants; 384 times indicated by midwives, 682 times indicated by women themselves and 197 times both the women and midwives responded positively. (Figure 1) [see Additional file 1] On the basis of history taking by telephone two weeks after delivery, 722 of the 869 women were excluded from participation. The majority of them, 635 women, were excluded because of a quick recovery, 13 women because of specific pathology, 49 women did not want to be randomized (clear treatment and therapist preference deviating from the study protocol), 12 women preferred not to participate on second thought, 10 women did not give informed consent for the intervention study and 3 women had moved outside the area where intervention was provided. History taking by telephone resulted in 147 home visits three weeks after delivery. Based on the home visits, 21 women were excluded: 15 women because of quick recovery, 2 women because of specific pathology, 1 woman because of a clear treatment preference and 3 women because of family or social reasons. Finally, from May 2001 until July 2003, 126 women were included in the intervention study.

### Baseline characteristics

Baseline status of the participants is given in Table 2. Both groups were highly similar in prognostic variables and baseline values of outcome measures.

### Follow-up

After randomization, 64 patients were assigned to the usual care group (UC group) and 62 to the experimental intervention group (EI group). In all patients, baseline data were complete. In the usual care group, for 56 patients (88%) data were available for all outcome measures at 12 weeks after delivery. In the experimental intervention group, the corresponding number was 58 (94%). In the usual care group 8 patients were lost to follow-up because of quick recovery within the first weeks after randomization and did not want the fill out the outcome assessments anymore (n = 3), lack of time (n = 4), or not motivated to fill out the outcome assessments (n = 1). In the experimental intervention group 4 patients were lost to follow-up because of family reasons (n = 1), discontinued intervention (n = 1) and quick recovery within the first weeks after randomization (n = 2). All patients who were lost to follow-up in both study groups were contacted by telephone.

### Compliance and co-intervention

In the experimental intervention group, two patients discontinued intervention. One woman because of lack of motivation and one woman because she could not endorse the basic principle of the experimental intervention (preferred a pain contingent treatment option on second thought). Nevertheless, the last mentioned patient completed all the outcome questionnaires and did not receive another treatment during the investigation period.

In the usual care group 22 women were treated by a physiotherapist, 4 women by a manual therapist, and 4 women were guided by their general practitioner. The other (n = 34) chose not to seek help for their complaints during the same period. Most reported reasons for not visiting a physiotherapist after all were:" too busy with housekeeping and care of the children," "progressing improvement after randomization" and "attention focused on the newborn baby, the low back and/ or pelvic girdle pain did not bother enough anymore to search for help."

In the experimental group 6 women used pain medication, one woman used an elastic belt and X-ray examined 2 women. In the control group 5 women used pain medication, 9 women an elastic belt and 5 women were examined by X-ray.

### Effectiveness of the experimental intervention compared to the usual care option

The outcome data were normally distributed. Measurement at 12 weeks after delivery showed a consistent difference in favor of the experimental intervention but only the primary outcome limitations in activities measured with the RDQ reached statistical significance (Table 3). More women in the experimental intervention group reported full recovery or very large improvement (74.1%) than women in the usual group (66.1%) did. The outcomes of the main complaint, pain, general health and the subscale fear-avoidance of the TSK favored the experimental intervention, but differences did not reach statistical significance.

Therefore, we performed a subgroup analysis to explore a possible different intervention effect in subgroups of women with severe limitations in activities at baseline. We expected that women with severe limitations in activities benefited more from the experimental intervention than from the usual care. We performed a median split on the baseline data of the RDQ and compared the both subgroups (baseline score RDQ < 13 versus ≥ 13). We found a statistically significant and clinically relevant difference of 4.3 points in favor of the experimental intervention in the subgroup RDQ ≥ 13 (Table 4). However we did not find a statistically significant difference in the subgroup RDQ < 13.

## Discussion

In our study, women with pregnancy-related pelvic girdle and/or low back pain benefited more from a tailor-made program with respect to biopsychosocial factors than from a usual care program on a pain contingent basis. At 12 weeks after delivery, we found a statistically significant difference in improvement between the two study groups in favor of the experimental intervention group that was clinically relevant (2 points) in limitations in activities measured with the RDQ. Other outcomes, such as global perceived effect, main complaint, pain, fear-avoidance and general health supported our main finding but did not reach statistical significance.

Although a substantial number of the participants of the cohort (n = 869) reported complaints and limitations in activities to the extent that treatment seems to be necessary during pregnancy, the prognosis (when not receiving treatment) is very good in the first weeks after delivery. For that reason the recruitment of women with persistent complaints and limitations in activities after delivery was lower than expected. We used the maximum of participants out of the cohort (n = 7526) and still included only 126 women. Nevertheless, we reached sufficient statistical power for the primary outcome, limitations in activities. But the smaller than planned study sample could have influenced the results of the other outcomes who were in line with the primary outcome but failed to reach statistical significance.

The favorable prognosis after delivery influenced probably also the improvement in both study groups. The recovery rates of both study groups were still relatively high, 12 weeks after delivery. The reasons why a relatively large number of women in the control group abandoned the current treatment option on second thought, leads to the suspect that the favorable prognosis and distraction of the mind by environmental factors (the new born baby and the change in family life) influenced the ask for help by a physiotherapist.

Longitudinal data of our cohort study (n = 7526) two weeks after delivery showed also a considerable drop in the number of women that reported pain in the lumbo-pelvic region. In literature there is some consensus about a good prognosis after delivery but not in the same extending as in our cohort. However, we could investigate the course of pregnancy-related pelvic girdle and/or low back pain more in detail and could combine it with the ask for help (physiotherapist) during pregnancy and after delivery in a very large group of women (n = 7526). Focusing only on pain did not answer our question if an intervention by a physiotherapist is advisable for whom and when, satisfactory. A reasonable number of women reported still some pain after delivery but they did not want to be referred to a physiotherapist anymore. Still having some pain was obviously not a deciding factor for many women to search for help. Other factors interfered such as a delay in becoming active again after delivery. Details about the enrollment out of the cohort into the trial showed that the start of the experimental intervention (three weeks after delivery) was reasonably well timed.

Subgroup analysis showed that women with moderate limitations in activities (score RDQ at baseline <13 points) improved quite well and nearly in the same extent in both study groups. However, in the subgroups with severe limitations in activities (score RDQ at baseline ≥ 13 points) the improvement favored the experimental intervention group compared to women in the usual care group. This finding underscored the main conclusion of the study clearly that women who received the experimental intervention benefited more from treatment than women who received usual care.

In general we did not deviate from the original study design, which was described independently of the study results [15]. At randomization, treatment groups were similar on demographics, patient characteristics and putative prognostic factors. To improve the transparency of the experimental intervention, we used a detailed protocol and standardized patient information by means of booklets.

Because of the nature of the experimental intervention, blinding of the therapists and women was not possible however the latter were kept naïve of the exact content of both intervention options. A clear treatment or therapist preference could influence responses regarding outcome measures. Therefore women who had a priori a treatment preference for a special treatment or therapist were excluded (n= 50).

Prior to the trial, we arranged interviews on an individual basis with women out of the cohort and groups' discussions with experienced physiotherapists and occupational therapists. The outcomes of those interviews were compared with literature findings and the outcomes of the group discussions. The answers of women about their perception of the contents of the current treatment approach and the patient-therapist relationship formed a sharp contrast with the view of the therapists about those topics. It made clear that worries about their condition in this population of women are not minor concerns. The worries referred to an unremitting source of anxiety about the etiology and prognosis. The degree of distress may contribute to avoidance of picking up the full range of activities again after delivery and the feeling that a wrong movement could lead to a serious problem, making individual concerns and worries an important target of care. These aspects and the contrast in perception between therapist and patient were important reasons to introduce, within the scope of a biopsychosocial approach, the theoretical concepts of self-management and fear-avoidance. The developed standardized patient information focused therefore on positive attitudes, an active response to the complaints and a physiological rationale about pain. It did not include information about a possible vulnerability of the spine or pelvis to damage and a relation with pain.

In general, women were willing to accept the responsibility to manage their own condition and solve their problems with guidance from the physiotherapists. They picked up the time contingent approach quickly. Nevertheless, there was some hesitation of a number of participating physiotherapists about their role in the intervention protocol during the educational training prior to the trial. Once they were be engaged with patients, they liked their supporting role of the women to create plans that will help her to achieve their goals. The shift from a pain contingent approach to a time contingent approach and the radical change in the character of the patient-therapist relationship seemed to be easier for the women than for the physiotherapists. Finally, both the women and the physiotherapists found the intervention protocol attractive and useful.

## Conclusion

The results of the trial supported the hypothesis. Although the biopsychosocial approach as a basis for intervention already is largely investigated in several musculoskeletal disorders and generated significant results, in the field of pregnancy-related pelvic girdle and/or low back pain it is a new approach. Women's individual worries and concerns about their condition were major targets in the experimental intervention. By adopting action plans as the most important act for goal setting, women's own values and the role of the therapist were included in the approach. The considerable drop of pain shortly after delivery influenced clearly the number of women that want to be referred to a physiotherapist. Because of the high level of improvement in both groups (even without treatment), it seems likely that the large improvement in the first weeks after delivery continued for a substantial deal in the first three months after delivery.

## Competing interests

The author(s) declare that they have no competing interests.

## Authors' contributions

CHGB: first author is involved in the design, data collection, statistical analysis, development of the experimental intervention, training the participating physiotherapist and the performance of the trial.

RAdeB: has made substantial contributions to conception of the study, obtained grants, participated in the design, coordination, statistical analyses, has made substantial contribution to the development of the experimental intervention and is involved in revising the article for important intellectual content.

PMJCW: participated in the design, has made substantial contribution to the development of the experimental intervention and is involved in revising the article for important intellectual content.

PL: participated in the statistical analyses and is involved in revising the article for important intellectual content.

JWSV: has made substantial contributions the development of the experimental intervention and choose of the measurements and is involved in revising the article for important intellectual content.

FS: participated as research-physician in this study and is involved in revising the article for important intellectual content.

JMB: participated in the design and statistical analyses and is involved in revising the article for important intellectual content.

GGME and PvdB: have made substantial contributions to conception, design and experimental intervention and revising the article critically for important intellectual content.

## Pre-publication history

The pre-publication history for this paper can be accessed here:



## Figures and Tables

**Table 1:** Timing of measures

	Baseline (about 3 weeks after delivery)	12 weeks after randomization
Baseline		
History taking	X	
Physical		
Examination:	X	
PBS	X	
Expectancy treatment result	X	
PCS	X	
BDI	X	
NEM	X	
PEM	X	
Primary outcomes		
RDQ	X	X
MC	X	X
GPE	X	X
Secondary outcomes		
MPQ(VAS)	X	X
TSK	X	X
SF-36	X	X
IPA	X	X
Compliance		X
Co-intervention		X
Medication		X
Additional medical consumption		X
Contents treatment		X

PBS = Pain Behavior Scale

GPE = Global Perceived Effect

MC = Main Complaint

MPQ = McGill Pain Questionnaire

RDQ = Roland Disability Questionnaire

TSK = Tampa Scale For Kinesiophobia

PCS = Pain Catastrophizing Scale

BDI = Beck Depression Inventory

NEM = Negative Emotionality Scale

PEM = Positive Emotionality Scale

SF-36 = Short-Form-36

IPA = Impact on Participation and Autonomy

**Table 2:** Baseline characteristics and measures according to treatment group

Variable	Usual Care n = 64	Experimental Intervention n = 62
Age (SD)	31.5 (3.1)	31.4 (3.6)
Localization of pain: (n/%)		
Lumbar spine	25 (39.1)	24 (38.7)
SI joints (one or two)	34 (53.1)	34 (54.8)
Symphysis	43 (67.2)	42 (67.7)
History of LB/PG pain ^⌂ ^(n/%)	43 (67.2)	44 (71.0)
Parity (SD)	2.1 (1.0)	2.3 (1.0)
Measures (SD)		
VAS (pain today) (0–100)	54.3 (17.5)	53.5 (19.1)
VAS (pain this week) (0–100)	59.2 (17.9)	57.0 (17.8)
RDQ (0–24)	13.5 (4.3)	13.3 (4.6)
IPA:		
Autonomy in self-care (0–28)	9.6 (5.7)	8.8 (5.1)
Mobility and leisure (0–20)	10.9 (3.7)	10.0 (4.7)
Family role (0–28)	15.4 (5.8)	14.4 (5.6)
Social relationships (0–24)	5.8 (3.3)	5.5 (3.4)
MC (VAS)^□ ^(0–100)	69.5 (17.5)	72.0 (15.7)
TSK:		
Total score (17–68)	35.7 (5.9)	32.9 (5.0)
Fear avoidance ^▶^(8–32)	18.8 (3.6)	17.0 (3.0)
Harm^▲ ^(5–20)	8.5 (2.2)	7.9 (2.3)
PCS (0–52)	12.2 (9.8)	11.2 (8.4)
BDI ^■ ^(0–54)	5.3 (4.9)	5.1 (4.2)
NEM (0–14)	3.3 (3.4)	2.4 (2.3)
PEM (0–11)	7.8 (2.6)	8.5 (2.4)
SF-36:		
General health (0–100)	54.6 (12.5)	57.8 (13.1)
Expectancy treatment (0–100)	79.0 (17.8)	78.3 (16.6)
PBS^◀ ^(0–8)	1.3 (1.3)	1.5 (1.6)

^■ ^mean BDI calculated without items 15,16,19

^□ ^first main complaint (MC) was selected

^▶^TSK subscale fear avoidance (items 1,2,9,10,13,14,15,17)

^▲^TSK subscale harm (items 3,5,6,7,11)

^◀ ^Pain behavior Scale filled out by research -physiotherapist after physical examination

^⌂ ^LB /PG pain = low back and/or pregnancy-related pelvic girdle pain

**Table 3:** Outcomes, mean change and differences between groups in outcome measures

Outcome measure	Outcome Usual Care N = 56 Mean SD	Outcome Experimental Intervention N = 58 Mean SD	Mean Change Usual Care N = 56 Mean SD	Mean change Experimental Intervention N = 58 Mean SD	Differences between groups Mean (95% CI) P-value
Primary outcomes					
RDQ (0–24)	6.8 (5.5)	4.5 (4.9)	6.7 (4.9)	8.7 (5.8)	-2.0 {-4.0;0.0} 0.05
MC (VAS) (0–100)*	26.8 (21.0)	21.0 (21.9)	41.5 (29.3)	50.8 (24.9)	-9.3 {-19.4;0.7} 0.07
GPE n / %#	37 (66.1)	43 (74.1)			-8 % {-24.2;8.2} 0.34
Secondary outcomes					
VAS (pain today)(0–100)	26.3 (19.9)	24.3 (24.1)	26.7 (22.5)	27.7 (25.4)	-1.0 {-9.9;7.9} 0.83
VAS (pain this week)(0–100)	29.6 (21.7)	25.3 (23.0)	29.2 (24.8)	30.3 (27.0)	-1.1 {-10.7;8.5} 0.82
IPA:					
Autonomy in self-care(0–28)	4.0 (4.6)	3.0 (4.3)	5.8 (5.1)	5.7 (5.4)	0.06 {-1.9;2.0} 0.95
Mobility and leisure (0–20)	6.3 (4.0)	5.6 (4.4)	4.6 (3.6)	4.4 (4.8)	0.14 {-1.5;1.8} 0.87
Family role (0–28)	9.2 (5.1)	7.8 (5.8)	6.3 (5.8)	6.3 (5.5)	0.04 {-2.1;2.1} 0.93
Social relationships (0–24)	4.4 (2.9)	4.0 (3.3)	1.0 (2.7)	1.5 (2.5)	-0.43 {-1.4;0.6} 0.39
TSK:					
Total score (17–68)	32.4 (5.6)	28.6 (6.0)	3.5 (5.8)	4.4 (5.8)	-0.9 {-3.0;1.3} 0.42
Fear Avoidance † (8–32)	16.2 (3.7)	13.5 (4.0)	2.6 (3.7)	3.5 (3.7)	-0.83 {-2.2;0.6} 0.24
Harm (5–20)‡	7.9 (2.4)	7.2 (2.3)	0.8 (6.0)	0.7 (2.2)	0.03 {--0.85;0.9} 0.95
SF 36:					
General health (0–100)	59.2 (13.7)	61.4 (14.0)	3.7 (13.8)	3.4 (9.5)	-0.39 {-4.8;4.0} 0.86

* First main complaint (MC) was selected, † TSK subscale fear avoidance (items 1,2 9, 10, 13, 14, 15, 17), ‡ TSK subscale harm (items 3, 5, 6, 7, 11), # ratings on a 7-point scale are dichotomized as improved (completely recovered and much improved) and not-improved (Slightly improved, not changed and slightly/ much/ vastly worsened)

**Table 4:** Subgroup analyses primary outcomes

Outcome measure	Outcome Usual Care Mean SD	Outcome Experimental intervention Mean SD	Mean change Usual Care Mean SD	Mean change experimental intervention Mean SD	Differences between groups Mean 95% P-value
Subgroup RDQ baseline < 13					
N	22	25			
Primary outcomes					
RDQ (0–24)	3.8 (3.0)	3.8 (3.6)	5.7 (3.4)	5.7 (4.5)	0.0002 {-2.4;2.3} 0.99
MC (VAS) (0–100) *	23.4 (23.5)	18.5 (19.3)	46.7 (30.0)	50.4 (19.5)	-3.3 {-17.9;11.4} 0.65
GPE n/ %#	16 (72.7)	20 (80.0)			-7.3 {-30.8;23.5} 0.55
Subgroup RDQ baseline ≥ 13					
N	34	33			
Primary outcomes					
RDQ (0–24)	8.7 (5.9)	5.5 (5.5)	6.4 (5.8)	10.7 (6.0)	-4.3 {-7.0;-1.5} 0.003
MC (VAS) (0–100) *	29.0 (19.2)	22.9 (24.7)	38.0 (28.9)	51.5 (28.5)	-13.4 {-27.4;0.6} 0.06
GPE n/ %#	21 (61.8)	23 (69.8)			-8.0 {-22.5;14.6} 0.49

* First main complaint (MC) was selected, # ratings on a 7-point scale are dichotomized as improved (completely recovered and much improved) and not-improved (Slightly improved, not changed and slightly/ much/ vastly worsened)
